# Improving the Experience of Doctors in Higher Psychiatry Training

**DOI:** 10.1192/bjo.2026.11404

**Published:** 2026-06-30

**Authors:** Magdalena Jimenez Cornejo, Sarah Tai, Lubna Anwar

**Affiliations:** North London Foundation Trust, London, United Kingdom

## Abstract

**Aims:**

1. To improve General Adult/Older Adult Higher training experience across North London Foundation Trust at 80% at 1 year.

2. To increase Higher trainee engagement with rotation evaluation survey to 80% at 1 year.

**Methods:**

We ran an initial survey amongst NLFT higher trainees, exploring their level of wellbeing in their current posts in 2024. Areas of improvement were identified and mappedusing a fish-bone diagram. During 2025, we held a series of iterative meetings, including with directors of medical education, training programmedirectors, higher trainees and reps to agree on concrete actions for improvement. We ran a second survey in 2025 to re-audit.

**Results:**

We achieved over a 80% satisfaction with the current SpR posts, however, re-audit survey was answered by fewer respondents than expected.

**Conclusion:**

An iterative process involving meetings with different stakeholders (trainees, clinical supervisors, training programme directors, etc.), openly discussing areas for improvement, and implementing small changes can improve trainee wellbeing. However, increasing participation in surveys remains a challenge.

